# Exploring the Evolutionary History and Phylogenetic Relationships of Giant Reed (*Arundo donax*) through Comprehensive Analysis of Its Chloroplast Genome

**DOI:** 10.3390/ijms25147936

**Published:** 2024-07-20

**Authors:** Lin Luo, Qi Qu, Hui Lin, Jiaming Chen, Zhanxi Lin, Ensi Shao, Dongmei Lin

**Affiliations:** Juncao Science and Ecology College, National Engineering Research Center of JUNCAO, Fujian Agriculture and Forestry University, Fuzhou 350002, China; 2210514017@fafu.edu.cn (L.L.); 52362043017@fafu.edu.cn (Q.Q.); ljuncao@163.com (H.L.); 15859907778@163.com (J.C.); lzxjuncao@163.com (Z.L.)

**Keywords:** *Arundo donax*, chloroplast genome, phylogenetic analysis, Arundionideae

## Abstract

Giant reed (*Arundo donax*) is widely distributed across the globe and is considered an important energy crop. This study presents the first comprehensive analysis of the chloroplast genome of giant reed, revealing detailed characteristics of this species’ chloroplast genome. The chloroplast genome has a total length of 137,153 bp, containing 84 protein-coding genes, 38 tRNA genes, and 8 rRNA genes, with a GC content of 39%. Functional analysis indicates that a total of 45 photosynthesis-related genes and 78 self-replication-related genes were identified, which may be closely associated with its adaptability and growth characteristics. Phylogenetic analysis confirmed that *Arundo donax* cv. Lvzhou No.1 belongs to the Arundionideae clade and occupies a distinct evolutionary position compared to other *Arundo* species. The findings of this study not only enhance our understanding of the giant reed genome but also provide valuable genetic resources for its application in biotechnology, bioenergy crop development, and ecological restoration.

## 1. Introduction

The giant reed (*Arundo donax* L.) is a perennial grass belonging to the Arundionideae within the Poaceae family. This sterile species is widely distributed in warm temperate and subtropical zones across the globe. Due to its high biomass production and remarkable adaptability to marginal lands, *A. donax* has gained recognition as a valuable resource for bioenergy, agronomy, and phytoremediation studies [1,2]. Previous investigations have indicated that *A. donax* is a polyploid plant with limited genetic diversity [3,4]. Despite extensive research on the genetic variability among *A. donax* populations over the past 20 years [4,5], the phylogenetic origin of this species remains unclear, primarily due to the lack of available genome information. Recently, chloroplast genomes have been utilized as “super barcodes” for species identification and phylogenetic analysis of angiosperms due to their conserved genetic composition and low nucleotide substitution rates [6,7]. Consequently, conducting a comprehensive genome analysis of *Arundo* species could provide valuable insights into the origins and evolution of this genus.

**Figure 1:**
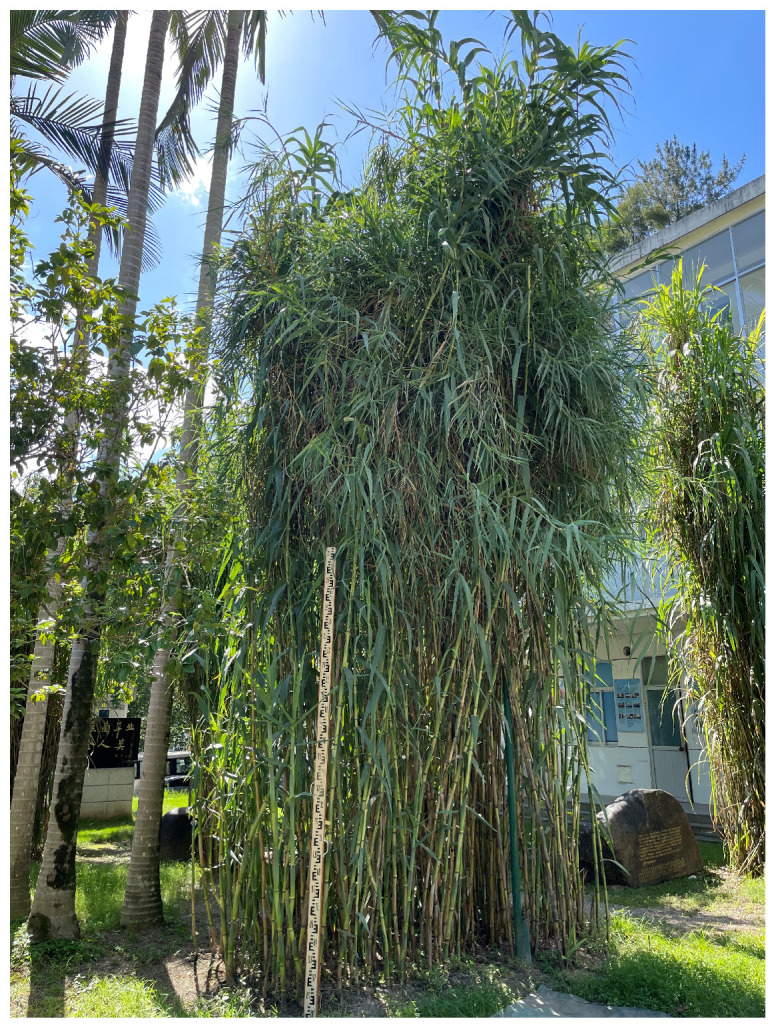
Individual canes of *A.donax* cv. Lvzhou No.1, reaching the height of 5 m, held by experts at JUNCAO.

In this study, we sequenced, assembled, and annotated the complete chloroplast genome of *A. donax* cv. Lvzhou No.1 (Figure 1) and conducted comparative genomic analyses with other Poaceae species. We performed simple sequence repeat (SSR) identification, codon usage bias analysis, and chloroplast genome comparative analysis on the chloroplast genomes of Arundinoideae downloaded from GenBank. Results showed that among the three *Arundo* species, *A. donax* had the highest number of SSRs, while *A. formosana* had the lowest. The chloroplast genome of *A. donax* contained 52 protein-coding genes, comprising a total of 16,172 codons. We compared the four boundary regions of chloroplast genomes in eight Arundinoideae species. Although the chloroplast genome structure and the number and sequence of genes are highly conserved among these species, structural variations in the contraction and expansion of IR boundaries were clearly observed. This study not only lays the foundation for understanding the chloroplast genome of *Arundo* but also provides data support for more comprehensive phylogenetic studies of the genus.

## 2. Results

### 2.1. Chloroplast Genome Features

**Figure 2:**
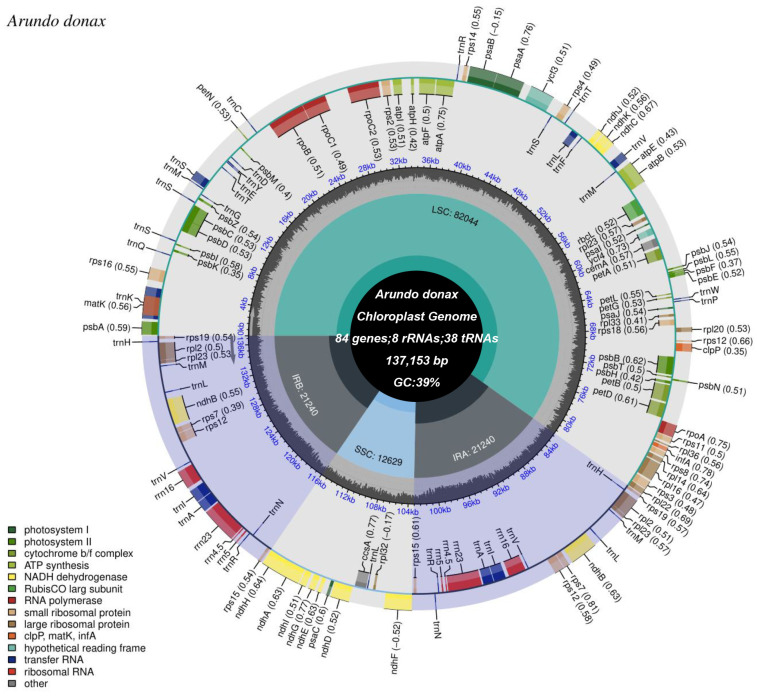
Circular map of the *A. donax* cv.lvzhou NO.1 chloroplast genome. From the center outward, the first track displays the small single-copy (SSC) sequence, the inverted repeat sequences (IRa and IRb), and the large single-copy (LSC) region. The GC content of the chloroplast genome is plotted on the second track. Genes are shown on the third track. The optional codon usage bias is indicated in parentheses following the gene names. Genes are color-coded based on their functional categories. The transcription directions of inner and outer genes are clockwise and counterclockwise, respectively. The functional classifications of the genes are displayed in the lower left corner.

The complete chloroplast genome of *A. donax* cv.No. 1 is 137,153 bp in length and consists of three distinct regions: a large single-copy (LSC) region of 82,043 bp, a small single-copy (SSC) region of 12,628 bp, and a pair of inverted repeat regions (IRa and IRb) totaling 21,241 bp (Figure 2, Supplementary Table S1). The GC content of the entire genome and its three distinct regions (LSC, SSC, and IR) are as follows: 39% for the whole genome, 37% for the LSC, 33% for the SSC, and 44% for the IR regions. Sequencing depth and coverage charts indicate the correct assembly of the chloroplast genome (Supplementary Figure S1).

Within the chloroplast genome, a total of 84 protein-coding genes, 38 tRNA genes, and 8 rRNA genes were identified (Supplementary Table S2). Additionally, 45 genes related to photosynthesis and 78 genes related to self-replication were identified (Table 1). Based on functional classification, the genes in the *A. donax* chloroplast genome can be categorized into photosystem I, photosystem II, cytochrome b/f complex, ATP synthase, NADH dehydrogenase, Rubisco large subunit, RNA polymerase (e.g., *rpoA*, *rpoB*), small ribosomal proteins (e.g., *rps2*, *rps4*), large ribosomal proteins (e.g., *rpl2*, *rpl14*), and molecular chaperones (e.g., *clpP*, *matK*, *infA*), among others. Hypothetical reading frames and transfer RNAs (tRNAs) also occupy a significant portion. Moreover, the inverted repeat regions contain 18 genes, including six protein-coding genes (*rps19*, *rpl2*, *rpl23*, *rps7*, *rps12*, and *rps15*), eight tRNA genes (*trnH-GUG*, *trnM-CAU*, *trnL-CAA*, *trnV-GAC*, *trnI-GAU*, *trnA-UGC*, *trnR-ACG*, and *trnN-GUU*), and four rRNA genes (*rrn16S*, *rrn23S*, *rrn4.5S*, and *rrn5S*). Additionally, one ndhF gene spans the SSC region (Figure 2). Furthermore, the *A. donax* cv.lvzhou NO.1 chloroplast genome annotates 11 cis-splicing genes (Supplementary Figure S2) and one trans-splicing gene (Supplementary Figure S3).

### 2.2. Simple Sequence Repeat (SSR) Analysis

**Figure 3:**
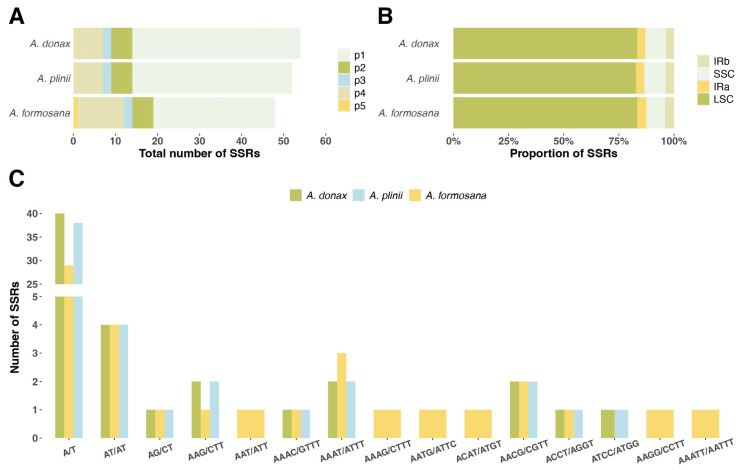
Distribution and quantity of SSRs in the chloroplast genomes of three *Arundo* species. (**A**) Total number of SSRs in *A. donax*, *A. plinii*, and *A. formosana* chloroplast genomes. The five types of SSRs (p1, p2, p3, p4, p5) are represented in different colors. (**B**) Proportion of SSRs located in different regions of the chloroplast genome: LSC (large single copy), SSC (small single copy), IRa (inverted repeat a), and IRb (inverted repeat b). (**C**) Number of SSRs of different types in the chloroplast genomes of *A. donax*, *A. plinii*, and *A. formosana*.

We analyzed five types of simple sequence repeats (SSRs) in the chloroplast genomes of three Arundo species. The distribution and quantity of SSRs (Figure 3, Supplementary Table S3). Among the three species, *A. donax* had the highest number of SSRs (52), while *A. formosana* had the fewest (48), and *A. plinii* had 52 SSRs (Figure 3B). Most of the detected SSRs were located in the LSC intergenic regions, with *A. plinii* having the lowest proportion in the LSC region at 83.3%. Two types of SSRs were detected in the inverted repeat regions (IRa, IRb) (Figure 3A). The most abundant type of SSR across all three Arundo species was the mononucleotide repeat A/T, with quantities ranging from 29 to 40. Notably, only *A. formosana* contained a pentanucleotide SSR (AAATT/AATTT). Additionally, other unique SSR types were detected in *A. formosana*, including AAT/ATT, AAAG/CTTT, AATG/ATTC, ACAT/ATGT, and AAGG/CCTT (Figure 3C).

### 2.3. Codon Usage Bias Analysis

**Figure 4:**
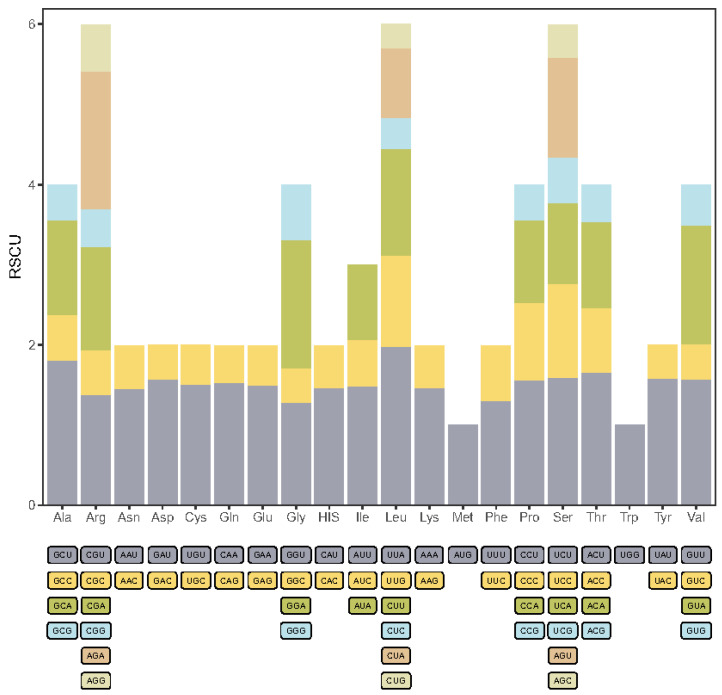
Stacked bar chart of relative synonymous codon usage (RSCU) in protein-coding genes of the *A. donax* chloroplast genome.

The *A. donax* chloroplast genome encodes a total of 35,753 codons. Codon usage bias analysis was performed on the protein-coding genes of the *A. donax* chloroplast genome, resulting in the relative synonymous codon usage (RSCU) values for 61 codons representing 20 amino acids (Figure 4, Supplementary Table S4). A total of 52 protein-coding genes were analyzed, yielding 16,172 codons, of which 32 codons had RSCU values greater than 1, indicating relatively high usage frequency. Among these high-frequency codons, 28 (87.5%) ended with an A/U base, suggesting a preference for A/U-ending codons in the *A. donax* chloroplast genome.

The AUU codon, which encodes isoleucine (Ile), was the most frequent, appearing 659 times. In contrast, the UGC codon, encoding cysteine (Cys), was the least frequent, with only 42 occurrences. Codons for leucine (Leu) included both the codon with the highest RSCU value (UUA, RSCU = 1.98) and the lowest (CUG, RSCU = 0.3). Additionally, two codons had an RSCU value of 1, indicating no codon preference: AUG for methionine (Met) and UGG for tryptophan (Trp).

### 2.4. Genomic Sequence Variation Analysis

**Figure 5:**
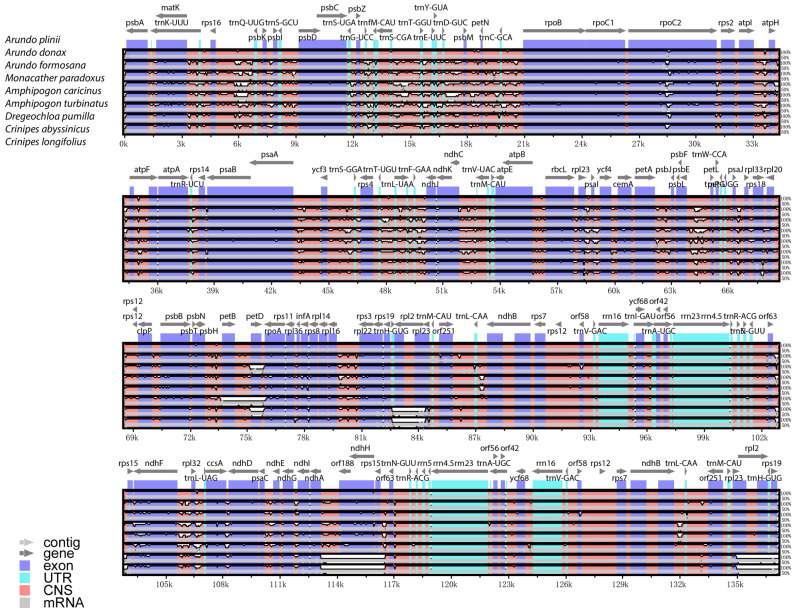
Sequence variation of chloroplast genomes in eight arundinoideae species. The top arrow indicates the transcription direction, the purple area indicates the protein-coding sequence (CDS), the red area indicates the conserved non-coding sequence (CNS), the gray area indicates the mRNA gene coding sequence, and the light green area indicates the tRNAs and rRNAs gene coding sequence. The X-axis represents the species class of the chloroplast genome, and the Y-axis represents the percentage within 50–100% homogeneity.

The mVISTA global alignment analysis of *A. donax* and its seven closely related species, using *A. plinii* as the reference chloroplast genome, reveals differences in chloroplast genome length and gene number among these species, though overall conservatism is high (Figure 5). Most genes, such as *psbA*, *rbcL*, and *ndhF*, are retained across these species, indicating their crucial roles in photosynthesis and chloroplast function. The genomic structures within the genus *Arundo* (e.g., *A. donax*, *A. plinii*, and *A. formosana*) are highly similar, with almost no significant differences in gene position and orientation. This high degree of conservation suggests that these species likely share a recent common ancestor and that their chloroplast genome structures have undergone minimal changes over evolutionary time.

*A. donax* is very similar to *A. plinii*, with only slight differences in the *atpI-atpH*, *atpF* genes, and non-coding regions. In contrast, the more distantly related *Crinipes* species, such as *C. abyssinicus* and *C. longifolius*, show greater differences compared to the *Arundo* species. Notable differences are observed in regions like the *rpl2* gene at approximately 84 kbp, genes like *orf188*, *ndhH*, and *rps15* around 114–116 k bp, and genes like *rpl2* and *rps19* around 135 k bp, where there are multiple blank segmented regions with sequence similarity below 50%.

Overall, the chloroplast genomes of the eight Arundinoideae species exhibit a certain degree of similarity, with greater variability in non-coding regions compared to coding regions. The LSC and SSC regions show more variation than the IR regions. 

### 2.5. Collinearity Analysis

**Figure 6:**
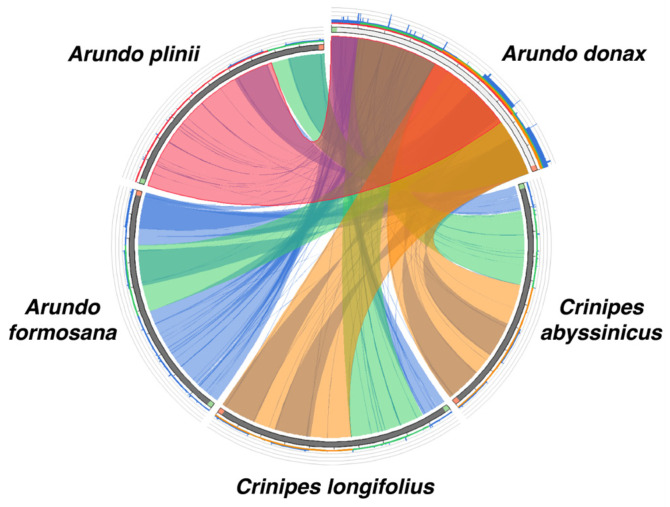
The comparison of similarity among five *Arundo* species. Sequences are connected with lines of different colors representing regions of similarity and similar lengths, as indicated by the color-coded scores in the histogram. Blank areas between the connecting lines indicate regions where no similarity exists between the two species.

The collinearity analysis compared the chloroplast genomes of *A. donax* with its four closely related species. The results (Figure 6) indicate a high similarity between *A. donax* and *A. plinii*, with over 75% collinearity, as evidenced by the numerous red lines connecting most regions without inversions. Surprisingly, *A. donax* shows the lowest similarity with the congeneric species *A. formosana*, with blue lines connecting most regions and a collinearity of less than 25%. The collinearity between *A. donax* and the *Crinipes* species (*C. abyssinicus* and *C. longifolius*) is higher than that between *A. donax* and *A. formosana*.

### 2.6. IR Boundary Comparison Analysis

**Figure 7:**
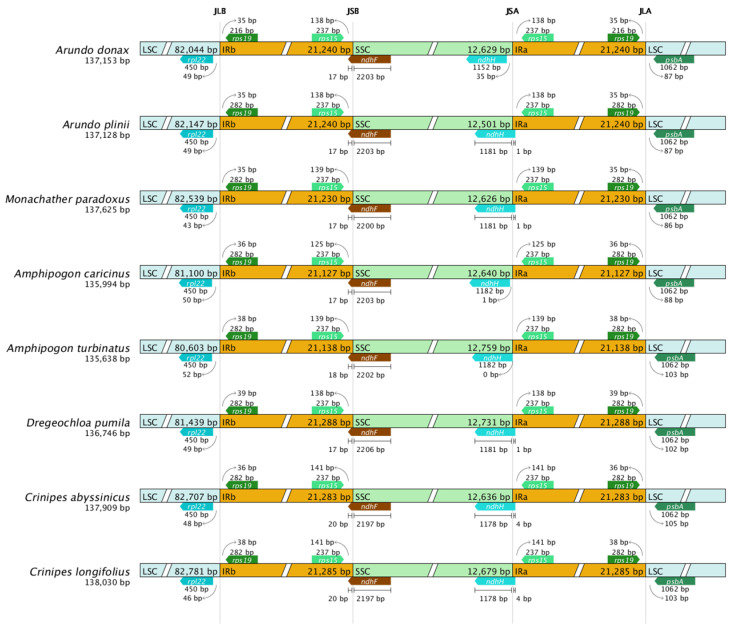
Comparison of IR region boundaries in chloroplast genomes of eight Arundinoideae species.

This study compared the four boundary regions of the chloroplast genomes of eight Arundinoideae species (Figure 7). Although the chloroplast genome structure, gene number, and sequences are highly conserved among Arundinoideae species, the contraction and expansion of the IR boundaries are structurally evident.

Due to the contraction of the IR region, the *ndhH* gene end is located 35 bp away from the JSA boundary in *A. donax*, 1 bp away in *Amphipogon caricinus*, exactly at the JSA boundary in *Amphipogon turbinatus*, while in other species, the *ndhH* gene spans the JSA boundary. The length of the ndhH gene also shows minor differences among species. For instance, the ndhH gene length in *A. donax* is 1152 bp, while in *Amphipogon caricinus* and *Amphipogon turbinatus*, it is 1182 bp. These slight length variations may be due to minor insertion or deletion events during evolution.

In all species, the *ndhF* gene spans the JSB boundary, indicating a similar degree of IR region expansion. Additionally, other boundary genes in each species do not cross the boundaries, and the extent of IR expansion into the LSC region is consistent. The psbA gene is located in the LSC region in all species, with no overlap between the LSC and IR regions. However, the position of the *psbA* gene within the LSC region varies, grouping the species into two categories. In the first group, the *psbA* gene is approximately 87 bp away from the JLA boundary in the first four sequences, while in the second group, the psbA gene is about 103 bp away from the JLA boundary in the last four sequences. Notably, the two *Amphipogon* species belong to different groups.

**Figure 8:**
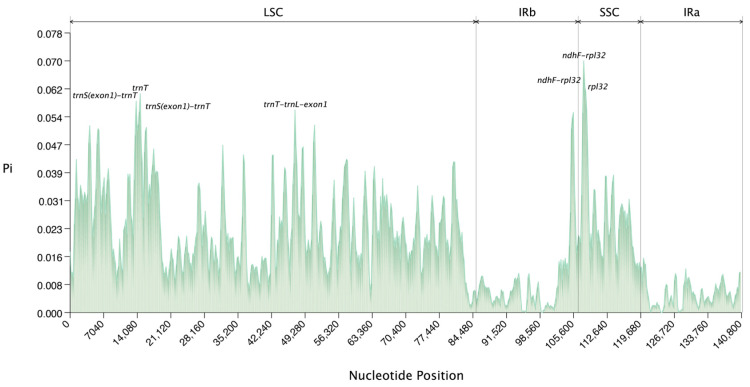
The nucleotide polymorphism (Pi) values of eight species of Arundinoideae were compared.

This study further analyzed the nucleotide polymorphism of eight species of Arundinoideae (Figure 8, Supplementary Table S5), detecting a total of 7367 polymorphic (segregating) sites in a 140,981 bp multi-sequence alignment. Seven highly variable regions were identified, with four located in the LSC and three in the SSC regions, which could serve as potential markers for species identification and molecular phylogenetic studies of Arundinoideae. Among the seven highly variable regions, two *ndhF-rpl32* and two *trnS(exon1)-trnT* regions were identified as highly variable, with the highest Pi value of 0.07 found in ndhF-rpl32. The Pi values for other variable regions are as follows: Pi(*ndhF-rpl32*): 0.0631, Pi(*rpl32*): 0.06131, Pi(*trnT*): 0.06095, Pi(*trnS(exon1)-trnT*): 0.05887, Pi(*trnT-trnL-exon1*): 0.05637, and Pi(*trnS(exon1)-trnT*): 0.05583. Overall, the single-copy regions exhibit higher variability compared to the inverted repeat regions.

### 2.7. Phylogenetic Analysis 

**Figure 9:**
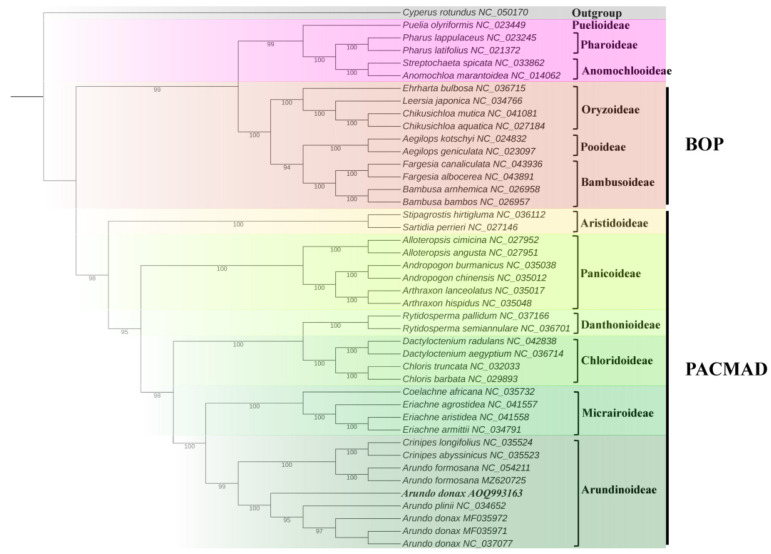
Phylogenetic tree containing chloroplast genome sequence of 43 grasses was constructed by maximum likelihood (ML) strategy using IQ-TREE with 10,000 ultrafast bootstraps.

To explore the phylogenetic relationships of *A. donax* with other species, a phylogenetic tree was constructed based on the alignment of chloroplast genome sequences from 42 Poaceae species, with *Cyperus rotundus* from the Cyperaceae family used as an outgroup to root the tree (Supplementary Table S6). The results show that the 42 Poaceae species clustered into 12 subfamily branches (Figure 9), with generally high bootstrap values, indicating a high degree of confidence in these phylogenetic relationships. In this study, all bootstrap values for the nodes were greater than 95%, suggesting that the chloroplast genomes of *Arundo* and its related species have maintained a high degree of consistency throughout their evolution.

*A. donax* cv. No. 1 along with six species of the genus *Arundo* and two species of the genus *Crinipes* (*C. abyssinicus* and *C. longifolius*), belongs to the Arundinoideae branch. These results represent the maximum likelihood phylogeny of previously fully plastid genomes of Arundinoideae species. The two *A. formosana* samples (MF035971 and MF035972 in Figure 9 of this study) were assigned to a separate clade, which includes the two *Crinipes* species [8]. This suggests that *A. formosana* may have diverged from the other *Arundo* species (*A. donax* and *A. plinii*).

Additionally, the origin and evolution of *A. donax* remain unclear. Our phylogenetic study indicates that *A. donax* cv. No. 1 occupies a distinct phylogenetic position compared to other *A. donax* varieties and *A. plinii* (Figure 9). This result suggests that *A. donax* cv. No. 1, collected from Fujian Province, China, may represent a variety with a different genetic origin from the other *A. donax* varieties included in this study.

## 3. Discussion

### 3.1. Chloroplast Genome Features

Chloroplast genome research holds significant importance for studies in plant taxonomy, evolutionary biology, and ecology [9]. By analyzing the chloroplast genome of *Arundo*, we can gain a better understanding of its photosynthesis mechanisms, genetic diversity, and adaptive evolution [10]. This research provides a foundation for investigating its photosynthetic efficiency, metabolic processes, and carbon fixation capacity [11]. The *Arundo* genus comprises six species, of which only *A. plinii* and *A. formosana* have had their chloroplast genomes sequenced and assembled, indicating that research on the *Arundo* genus is limited. The chloroplast genome of *A. donax* is approximately 137 kb in size, which falls within the normal range for chloroplast genomes of the *Arundo* genus in the Poaceae family [12]. Furthermore, its GC content distribution is similar to that of other Poaceae plants, such as the chloroplast genome of *Oryza minuta* [13]. The chloroplast genome encodes genes with various functions, including photosynthesis proteins, RNA polymerases, ribosomal proteins, as well as multiple transfer RNA and ribosomal RNA. The arrangement and transcriptional direction of these genes are crucial for the regulation of gene expression, and their characteristic arrangement helps us understand the spatial and temporal specificity of gene expression. Notably, the genes *ycf1*, *ycf2*, and *ycf15* were not annotated in the chloroplast genome, suggesting a possible loss. Previous reports on Poaceae chloroplast genomes also noted the loss of *ycf1* and *ycf2* genes [14,15]. Additionally, we identified the trans-spliced gene *rps12*, which, like in most species, comprises three exons [16].

By analyzing the chloroplast genome, we can also identify key genes related to photosynthesis, protein synthesis, and stress response (such as photosystem I, photosystem II, and ATP synthase), which can be targeted for gene editing to enhance the photosynthetic efficiency or increase the tolerance of *A. donax* to environmental stresses such as drought and salinity. Previous studies have successfully achieved efficient base editing in the chloroplasts of lettuce, rapeseed, and rice through the development and application of TALE-adenine base editors (TALE-ABEs) and DddA-derived cytosine base editors (DdCBEs) [17,18]. Furthermore, recent research has utilized an artificial targeting system to relocate the chloroplast cytochrome b6f complex (PETD protein), NADH dehydrogenase A (NDHA), and NADH dehydrogenase B (NDHB) to chloroplasts, thereby enhancing photosynthetic efficiency and stress resistance in plants under environmental pressures, which in turn increases plant yield [19]. Molecular chaperones (such as *clpP*, *matK*, and *infA*) play a critical role in protein folding and repair, and studying these genes can improve the plant’s ability to adapt to environmental stress, allowing them to survive and thrive in damaged ecosystems. Existing research has demonstrated that *A. donax* performs well on marginal lands, showing good adaptability and stability [20]. As a high-biomass-energy crop, it exhibits high tolerance to cadmium, chromium, copper, nickel, and lead, and has the ability to accumulate these heavy metals under in vitro conditions [21]. By breeding or engineering plants containing these key genes (photosynthesis-related genes and self-replication-related genes), we can enable them to grow in polluted soils and water bodies, absorbing and degrading pollutants to purify the environment. Moreover, we can utilize these genes to enhance root growth, which can improve soil structure and nutrient cycling, thereby restoring soil health.

Additionally, by introducing or overexpressing these key genes, it is possible to develop transgenic crops with superior traits, such as faster growth rates and enhanced resistance to pests and diseases. We can delve deeper into understanding and optimizing the energy metabolism pathways of plants to increase the energy conversion efficiency of bioenergy crops. By regulating genes related to energy storage, we can increase the sugar content of *A. donax*, thereby enhancing its potential as a biofuel feedstock [22]. For instance, enhancing the expression of genes involved in sugar synthesis and accumulation can allow *A. donax* to accumulate more sugars within a shorter growth period, increasing its value as a feedstock for bioethanol production.

Furthermore, due to its high cellulose content and good enzymatic hydrolysis efficiency, it can significantly increase ethanol yield (up to 82.59 ± 7.42%) after ultrasound-assisted alkaline pretreatment [23]. This pretreatment method effectively disrupts plant cell wall structures, releasing more fermentable sugars and thereby increasing ethanol yield. Research also indicates that *A. donax* not only has significant advantages in ethanol yield but also its high cellulose content makes it an ideal raw material for biofuel production. Through further genetic engineering and optimization, it is possible to increase its cellulose and sugar content, reduce production costs, and improve energy conversion efficiency.

Analyzing the codon usage bias of the chloroplast genome can reveal the coding preferences for specific amino acids, reflecting the adaptive evolution of genetic coding. Codon bias may influence the translation efficiency and accuracy of proteins, thereby affecting plant growth and development [24]. The high-frequency codons in the *A. donax* chloroplast genome prefer A/U base endings, consistent with studies on orchid chloroplast genomes [25]. Simple sequence repeats (SSRs) have a high mutation rate and are prone to slippage during replication, leading to variations in the number of repeat units and consequently microsatellite length variations [26]. The presence of SSRs may be associated with biological functions such as gene regulation and genome recombination, and may also affect gene transcription and expression [27]. SSRs can serve as genetic markers for species identification, phylogenetic analysis, and population genetics studies [28]. The distribution of SSRs in the IR region is relatively conserved across the three species of the *Arundo* genus, with two types of SSRs, p2 (TA) and p4 (AACG/TCGT), found in the IR region. Dinucleotide repeats are located in the intergenic spacer (IGS) between trnI-CAU and trnL-CAA, while tetranucleotide repeats are located within the rrn4.5 gene. Most SSRs are distributed in the intergenic spacer regions (IGS), with a few found within genes. Both *A. donax* and *A. plinii* have the highest number of SSRs within the *rpoC2* gene, which encodes the chloroplast RNA polymerase C2 subunit, a component of the RNA polymerase core enzyme involved in chloroplast gene transcription [29]. The *rpoC2* gene is highly conserved across different species, but the SSR sequences within it may exhibit high variability. This phenomenon suggests that while the overall structure and function of the gene remain unchanged, SSR variability can provide diversity and adaptability. This study fills a significant gap in the research on SSR loci in the genus *Arundo*, providing a foundation for developing molecular markers and identifying species within this genus. With the development of SSR markers in various species [30], we can leverage chloroplast genome information to perform marker-assisted selection, thereby accelerating traditional breeding processes and developing new varieties better suited to specific environmental conditions.

Given the richness of SSRs in the *rpoC2* gene in both *A. donax* and *A. plinii*, these SSRs can serve as important molecular markers for genetic diversity studies and phylogenetic analysis of these two species. Only in the *A. formosana* chloroplast genome were pentanucleotide SSRs found, along with some unique SSR types, which can serve as molecular markers for identifying *A. formosana*. In previous research, 8364 SSRs were discovered in the leaf transcriptome of *A. donax* [31]. It was found that SSRs are more abundant in the intergenic non-coding regions than in the gene-coding regions, similar to the distribution of SSRs in the chloroplast genome found in this study. Overall, the types of SSRs are similarly distributed in *A. donax* and *A. plinii,* but differ significantly compared to *A. formosana.*

### 3.2. Comparative Genomic Analysis

There are some non-coding regions in the chloroplast genome that may play a role in regulating gene expression [32,33]. The order of genes and the direction of transcription are also crucial for the regulation of gene expression, and their specific arrangement helps us understand the spatiotemporal specificity of gene expression [34]. By comparing genome sequence differences among different species, we can observe significant sequence differences between different genera. These differences are mainly reflected in non-coding regions and some gene-coding regions, possibly due to mutations and recombination events during evolution [35].

Collinearity analysis can identify the gene arrangement order between different chloroplast genomes, assess important conserved and variable regions, and aid in understanding genome evolution [36]. Further analysis of the genus Arundo and the closely related genus *Crinipes* reveals a strong collinearity relationship between species of the genus *Arundo* (*A. donax* and *A. plinii*), with consistent gene arrangement and location, further supporting their close relationship. However, the collinearity relationship with more distantly related species is weaker, with some genes undergoing rearrangement or loss. These genome rearrangement events may be the result of plants adapting to different environmental pressures, reflecting divergent evolutionary paths. 

Nucleotide polymorphism analysis indicates that the IR region is more conserved, making it an ideal site for transgene integration in the chloroplast genome [10]. The boundaries of the IR region vary in the chloroplast genomes of different species, possibly due to mutations in some genes. These changes may affect the stability of the chloroplast genome, gene replication, and expression [37]. Through comparative analysis and CPGView sequence examination, we found that the IR region of *A. formosana* was misassembled, with unequal sequence lengths in the two IR regions, preventing reverse complementarity (details in Supplementary Table S7). Therefore, IR boundary analysis excluded the *A. formosana* species.

The chloroplast genomes of the Arundinoideae are conserved in terms of gene composition and structure, with no significant expansion or contraction observed in the IR regions. Minor differences were observed in the *psbA* and *ndhH* genes at the IR region boundaries in the comparative analysis of the Arundinoideae. The *psbA* gene encodes the D1 protein, which is the core reaction center protein of photosystem II [38]. The *ndhH* gene encodes the NADH dehydrogenase H subunit, which is part of the NADH dehydrogenase complex [39]. Both genes play key roles in photosynthesis and are highly conserved. Their high degree of conservation is likely due to the critical importance of their functions. Any significant alterations to these photosynthesis-related genes could negatively impact the efficiency of photosynthesis in plants, leading to their high conservation throughout evolution [40].

Studies have shown that in the leaf cross-sections of *A. donax*, chloroplasts are only present in mesophyll cells and absent in bundle sheath cells, indicating that it is a C3 grass [41]. However, compared to other C3 species, *A. donax* has a very high photosynthetic capacity, comparable to that of C4 bioenergy grasses [42]. Further in-depth studies on these key photosynthetic genes may reveal the mechanisms underlying the exceptionally high photosynthetic capacity of *A. donax*.

### 3.3. Phylogenetic Implications and Biogeographic History

Phylogenetic analysis helps to elucidate the evolutionary relationships and taxonomic status among plants. As a plant with significant ecological value, clarifying the phylogenetic position of *A*. *donax* provides guidance for the conservation and utilization of related species. Phylogenetic analysis can provide reliable molecular evidence for the classification of *A. donax* and offer a scientific basis for its ecological protection.

Gramineae (Poaceae) plants can be divided into two major clades: the BOP clade (including Bambusoideae, Oryzoideae, Pooideae, etc.) [43] and the PACMAD clade (including Panicoideae, Arundinoideae, Chloridoideae, etc.) [44]. This study found that the Arundinoideae has a closer phylogenetic relationship with the Micrairoideae within the PACMAD clade, and they form a major branch together. This finding is consistent with previous studies, which have identified the Micrairoideae as the sister group to Arundinoideae [8]. Based on the collected data on molecular analysis, chromosome number, epidermal cell size, and chlorophyll content, previous studies proposed the hypothesis that *A. donax* might be a polyploid derived from *A. plinii* [4]. The results of this study corroborate this hypothesis, showing that *A. donax* is most closely related to *A. plinii* in the phylogenetic tree.

The phylogenetic tree shows the genetic consistency of *A. donax* samples collected from different regions (e.g., OQ993163 from East Asia, MF035972 and MF035973 from North America, and NC037077 from the Mediterranean region), further demonstrating its clonal reproduction characteristics and lack of genetic diversity. Similarly, previous studies have shown that *A. donax* collected from the United States, ranging from California to South Carolina and Florida, did not exhibit molecular genetic variation [45]. *A*. *donax* has a robust rhizome system and rapid growth capability, making it highly competitive in wetland and riparian areas, thus demonstrating high ecological adaptability and the ability to grow under different environmental conditions [46]. However, most *A*. *donax* are sterile due to ovule development failure and primarily reproduce asexually, which allows them to spread rapidly and occupy new habitats [47]. Therefore, asexual reproduction is also one of the reasons for its low genetic diversity.

Additionally, the sample OQ993163 collected from Fujian, East Asia, is located at the base of the *A. donax* branch (MF035972, MF035973, NC037077 sampled from Italy) within the Arundinoideae subfamily in the phylogenetic tree. This suggests that *A. donax* in East Asia may be the origin of the species in North America and the Mediterranean region. Previous studies have yielded similar results, indicating that *A. donax* may have originated in East Asia [47]. Unlike species that spread north–south, *A. donax* likely originated in East Asia and then spread to the Middle East and the Mediterranean region, areas with relatively consistent climates [48]. Moreover, because of its asexual reproduction through rhizomes and stem fragments, it can rapidly clone and spread [49]. This adaptability has enabled it to spread and occupy new habitats globally with minimal ecological adaptation [50]. *A. donax* was artificially introduced to North America in the early 19th century, primarily for erosion control [51]. Samples collected from St. Louis, North America (MF035972, MF035973), indicate its successful colonization and spread on the North American continent, becoming an invasive species in the region. Throughout history, human activities have also played a significant role in the global spread of *A. donax*. It has been used for various purposes, such as paper production, musical instrument making, and agriculture, thereby promoting its dissemination worldwide [52]. By combining our results with other studies, we propose possible dispersal pathways and ecological adaptation strategies of *A. donax*, explaining its occurrence in different geographical regions and its ecological adaptability.

Future research can further elucidate the functional genes in the chloroplast genome of *A. donax* and their expression regulation mechanisms. Using plastid transformation technology, important agronomic traits can be introduced into the chloroplast genome of *A. donax* to further improve its agronomic characteristics. Additionally, combining research on nuclear and mitochondrial genomes can provide a comprehensive analysis of the genetic structure and evolutionary history of *A. donax*.

## 4. Materials and Methods

### 4.1. Plant Materials, Chloroplast DNA Extraction, Sequencing

The *Arundo donax* cv. Lvzhou NO.1 specimen is preserved at the China National Engineering Research Center of JUNCAO Technology of Fujian Agriculture and Forestry University (http://www.juncao.org, accessed on 25 June 2023, Lin Hui, lzxjuncao@163.com), with voucher number Juncao 20061009. For this study, fresh mature leaf samples of approximately 25 g were collected from the plant grown at a germplasm resource nursery in Fujian Province, China. Chloroplast DNA was extracted using the modified CTAB method [53]. The quality and quantity of the extracted DNA were measured using a Qubit^®^ 3.0 Fluorometer (Thermo Fisher Scientific, Waltham, MA, USA, Cat Q33216). Approximately 10 μg of isolated chloroplast DNA was sheared and ligated with adapters. DNA libraries were amplified following Illumina’s sample preparation instructions, and 2 × 150 bp paired-end reads were generated using the Illumina HiSeq 4000 platform(Illumina, San Diego, CA, USA). The raw data were preprocessed by quality control of whole-genome sequencing data using the fastp tool to remove low-quality and adapter sequences, ensuring the accuracy of subsequent analyses [54].

### 4.2. Chloroplast Genome Assembly and Annotation

The chloroplast genome of *A*. *donax* cv. Lvzhou NO.1 was assembled using the “get_organelle_from_reads.py” script from the GetOrganelle software (v1.7.7.0). This process leveraged the Bowtie2, BLAST, and SPAdes packages and employed a hashing algorithm for the assembly. The assembly results were visualized and evaluated using Bandage software (v 0.8.1), followed by manual connection of non-target contigs/scaffolds [55,56]. Subsequently, the “get_organelle_from_assembly.py” script was utilized to clean the assembly graph, yielding the complete chloroplast genome of it. The assembled sequences were imported into the online tool CPGAVAS2 (http://47.96.249.172:16019/analyzer/home, accessed on 8 March 2023) for gene annotation [57]. The annotation results were further refined using Geneious (v 9.0.2) software to annotate gene structures and functions. The corrected annotation files were imported into CPGView (http://www.1kmpg.cn/cpgview/, accessed on 9 March 2023) for visualization to obtain a circular chloroplast genome map [58].

### 4.3. Analysis of Repeat Sequences

Simple sequence repeats (SSRs) in the chloroplast genome were predicted to analyze their distribution and characteristics. In this study, the online tool MISA (https://webblast.ipk-gatersleben.de/misa/, accessed on 3 May 2024)) was utilized to import the *A*.*donax* chloroplast genome fasta file. Parameters were set to determine the minimum repeat numbers for simple sequence repeats as follows: mononucleotide, dinucleotide, trinucleotide, tetranucleotide, pentanucleotide, and hexanucleotide were set to 10, 5, 4, 3, 3, and 3, respectively. This analysis provided information on the types and quantity of simple sequence repeats in the chloroplast genome.

### 4.4. Analysis of Codon Usage Bias

We conducted a statistical analysis of codon usage bias in protein-coding genes within the chloroplast genome of reeds to understand their preferences and patterns. Relative synonymous codon usage (RSCU) was calculated to assess codon bias. Firstly, PhyloSuite software (v 1.2.3) [59] was employed to extract the coding sequence (CDS) of protein-coding genes from the *A. donax* chloroplast genome, followed by the removal of sequences that did not meet the research criteria. It was ensured that there were no duplicate gene sequences, sequences with a length greater than or equal to 300 bp, or sequences with a number of bases divisible by 3, and that each sequence began with a start codon and ended without a stop codon. R scripts were used to calculate the RSCU values for each codon, and the results were visualized using the ggplot2 package to generate stacked bar plots [60].

### 4.5. Chloroplast Genome Visualization and Sequence Divergence Analysis

Use the mVISTA online analysis tool (https://genome.lbl.gov/vista/mvista/submit.shtml/, accessed on 8 May 2024) with *A*. *plinii* as a reference to compare the chloroplast genome sequences of different individuals within the Arundinoideae, analyzing differences and variations between the sequences. To facilitate this analysis, Python (v3.7.6) scripts were employed to systematically convert chloroplast gene annotations from GenBank files of the aforementioned species into the appropriate mVISTA format. Leveraging *A*. *plinii* as the reference sequence, the Shuffle-LAGAN algorithm [61] was specifically chosen to facilitate visual comparison of chloroplast genome variation regions. Additionally, Circoletto [62] was utilized to assess the similarity between *A*. *donax* and closely related species within the Arundinoideae, with an E-value threshold established at 1 × 10^−10^.

For a comprehensive examination of chloroplast genome characteristics within the Arundinoideae, data from eight species were incorporated into CPJSdraw (v1.0.0), guided by their phylogenetic relationships [63]. This facilitated the alignment of the inverted repeat (IR) regions and the creation of diagrams illustrating IR boundary comparisons. Subsequently, the boundaries of IR regions across different species were meticulously compared to elucidate any disparities or variations.

To delve deeper into nucleotide polymorphisms within chloroplast genome sequences, alignment was conducted using MAFFT(v 7.520), followed by SNP analysis using DnaSP (v 6.12.03) [64]. Nucleotide polymorphisms, represented by Pi values, were calculated employing a sliding window approach, with a window size not exceeding 600 bp and a sliding interval of 200 bp. These findings were graphically presented using line graphs, aiding in the comprehension of their implications for population genetic structure.

### 4.6. Phylogenetic Analysis

We selected representative species in the phylogenetic tree that cover the major subfamilies and genera within the Poaceae family, specifically choosing representative species from the main branches, PACMAD and BOP, within the Poaceae. This selection includes representative species from the nine major subfamilies to ensure a comprehensive analysis. Next, we obtained 42 Poaceae chloroplast genomes from GenBank and combined them with the chloroplast genome of *A*. *donax* cv. Lvzhou NO.1, obtained in this study, for phylogenetic analysis. Genome sequences were aligned using MAFFT (v 7.520) [65], and conserved domains were selected using Gblocks (v 0.91b) [66]. The chloroplast genome of *C. rotundus* was used as the outgroup for the phylogenetic analysis. ModelFinder [67], implemented in IQTREE (v 2.2.0) [68], was used to determine the best partition scheme and model for the analysis. According to the Bayesian Information Criterion (BIC), GTR+F+G4 was selected as the best-fit model. Maximum likelihood analysis with 10,000 ultrafast bootstrap replicates [69] was conducted using IQTREE. The resulting tree was visualized and annotated using Interactive Tree of Life (http://itol.embl.de, accessed on 15 May 2024).

## 5. Conclusions

In this study, we sequenced, assembled, and annotated the complete chloroplast genome of *A. donax*, revealing a genome length of 137,153 bp. It comprises three distinct regions: a large single-copy (LSC) region of 82,043 bp, a small single-copy (SSC) region of 12,628 bp, and a pair of inverted repeat (IR) regions totaling 21,241 bp. The overall GC content is 39%, with 37% in LSC, 33% in SSC, and 44% in IR regions. The genome contains 84 protein-coding genes, 38 tRNA genes, and 8 rRNA genes. Comparative analyses identified 52 simple sequence repeats (SSRs) predominantly in the LSC region. Phylogenetic analysis positioned *A. donax* within the Arundinoideae subfamily, closely related to *A. plinii* and *A. formosana*. The study highlights high nucleotide polymorphism in single-copy regions compared to IR regions. Our findings provide valuable insights into the genomic structure, phylogeny, and evolutionary history of *A. donax*, contributing to future research and applications in biotechnology, bioenergy, and ecological restoration.

## Figures and Tables

**Table 1:** Genetic composition of chloroplast genome of *A. donax*.

Category	Gene Group	Gene Name
Photosynthesis	Subunits of photosystem I	*psaA*, *psaB*, *psaC*, *psaI*, *psaJ*
	Subunits of photosystem II	*psbA*, *psbB*, *psbC*, *psbD*, *psbE*, *psbF*, *psbH*, *psbI*, *psbJ*, *psbK*, *psbL*, *psbM*, *psbN*, *psbT*, *psbZ*
	Subunits of NADH dehydrogenase	ndhA*, ndhB*(2), *ndhC*, *ndhD*, *ndhE*, *ndhF*, *ndhG*, *ndhH*, *ndhI*, *ndhJ*, *ndhK*
	Subunits of cytochrome b/f complex	*petA*, petB*, petD*, *petG*, *petL*, *petN*
	Subunits of ATP synthase	*atpA*, *atpB*, *atpE*, atpF*, *atpH*, *atpI*
	Large subunit of rubisco	*rbcL*
Self-replication	Proteins of large ribosomal subunit	*rpl14*, rpl16*, rpl2*(2), *rpl20*, *rpl22*, *rpl23(3)*, *rpl32*, *rpl33*, *rpl36*
	Proteins of small ribosomal subunit	*rps11*, rps12**(2), *rps14*, *rps15(2)*, rps16*, *rps18*, *rps19(2)*, *rps2*, *rps3*, *rps4*, *rps7(2)*, *rps8*
	Subunits of RNA polymerase	*rpoA*, *rpoB*, *rpoC1*, *rpoC2*
	Ribosomal RNAs	*rrn16S(2)*, *rrn23S(2)*, *rrn4.5S(2)*, *rrn5S(2)*
	Transfer RNAs	trnA-UGC*(2), *trnC-GCA*, *trnD-GUC*, *trnE-UUC*, *trnF-GAA*, *trnG-GCC*, *trnH-GUG(2)*, trnI-GAU*(2), trnK-UUU*, *trnL-CAA(2)*, trnL-UAA*, *trnL-UAG*, *trnM-CAU(4)*, *trnN-GUU(2)*, *trnP-UGG*, *trnQ-UUG*, *trnR-ACG(2)*, *trnR-UCU*, trnS-CGA*, *trnS-GCU*, *trnS-GGA*, *trnS-UGA*, *trnT-GGU*, *trnT-UGU*, *trnV-GAC(2)*, trnV-UAC*, *trnW-CCA*, *trnY-GUA*
Other genes	Maturase	*matK*
	Protease	*clpP*
	Envelope membrane protein	*cemA*
	c-type cytochrome synthesis gene	*ccsA*
	Translation initiation factor	*infA*
Genes of unknown function	Conserved hypothetical chloroplast ORF	ycf3**, *ycf4*

Note: Gene*: Gene with one introns; Gene**: Gene with two introns; Gene (n): Number of copies of multi-copy genes, where "n" represents the number of gene copies.

## Data Availability

The genome sequence data support the results in this work are available in GenBank of NCBI at (http://www.ncbi.nlm.nih.gov, accessed on 25 June 2023) under the accession No. OQ993163. The associated BioProject, SRA, and Bio-Sample numbers are PRJNA974205, SRR24693295 and SAMN35174953, respectively. The sequence data utilized in this study can be found in Supplementary Material.

## Supplementary Materials

The following supporting information can be downloaded at: https://www.mdpi.com/article/10.3390/ijms25147936/s1.
