# Supplementary material for: Exploring the Evolutionary History and Phylogenetic Relationships of Giant Reed (Arundo donax) through Comprehensive Analysis of Its Chloroplast Genome
Source: Int J Mol Sci. 2024 Jul 20;25(14):7936. doi: 10.3390/ijms25147936 (PMC11277011; doi:10.3390/ijms25147936)
Supplement: Supplementary file 1 [file ijms-25-07936-s001.zip › ijms-3084387-Supplementary/Supplementary-figures.pdf]

Supplementary Materials

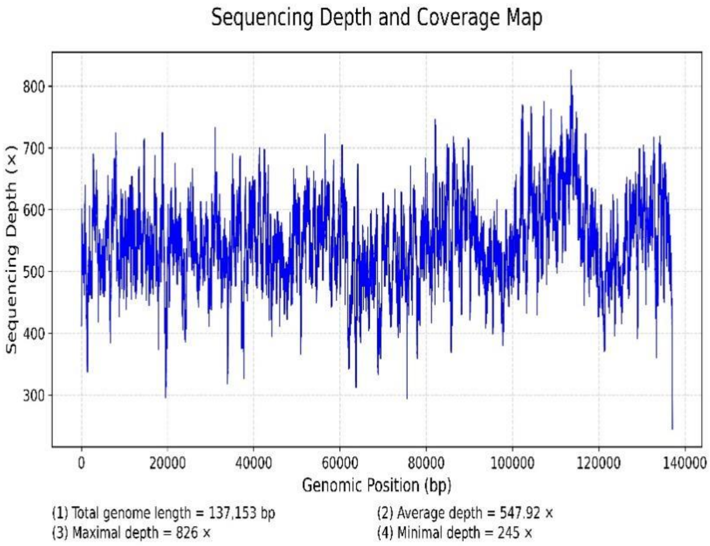

Supplementary Figure S1: Sequencing Depth and Coverage Map of Chloroplast Genome

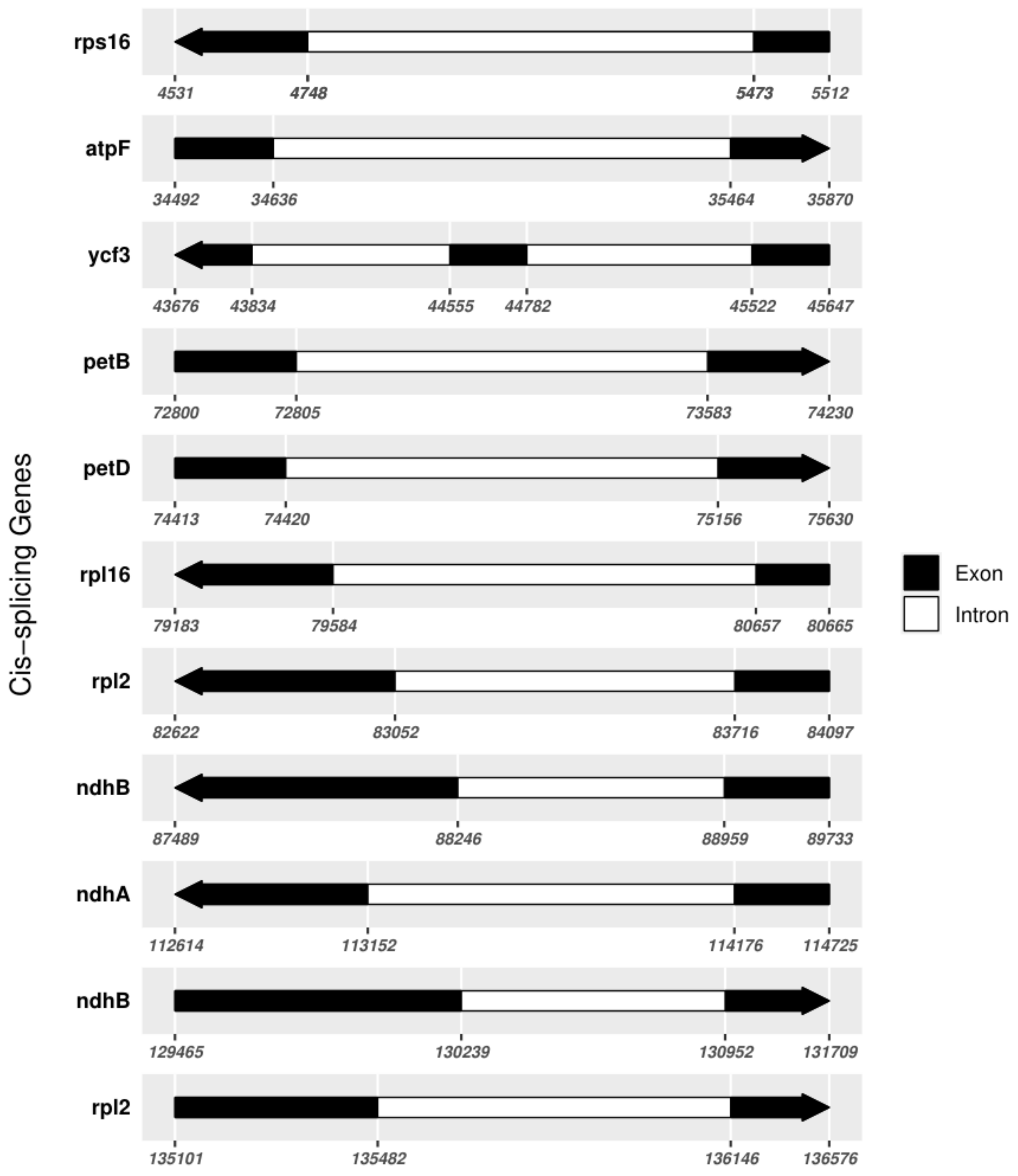

Supplementary Figure S2: Cis-Splicing Genes in the Chloroplast Genome

The diagram illustrates the cis-splicing genes in the chloroplast genome. Exons are shown in black, introns are shown in white. Arrows indicate the transcription direction of the genes. Please refer to the attached figure for detailed information.

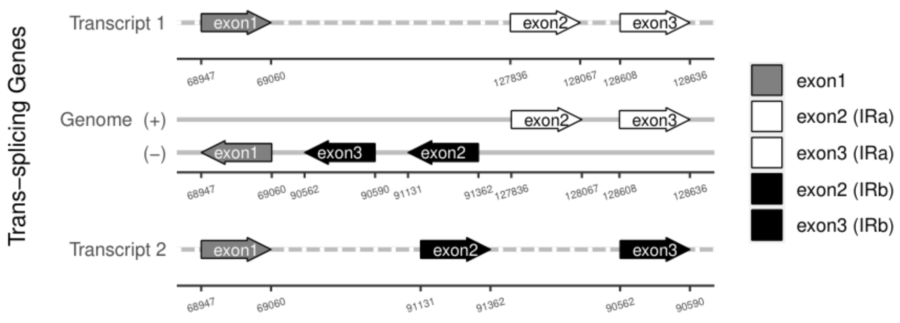

Supplementary Figure S3: Trans-Splicing Gene in the Chloroplast Genome
The diagram illustrates the trans-splicing gene rps12 in the chloroplast genome, showing two transcripts of the rps12 gene. It has three exons, with two exons duplicated as they are located in the IR region. Please refer to the attached figure for detailed information.
